# AKT/FOXO1 axis links cross-talking of endothelial cell and pericyte in TIE2-mutated venous malformations

**DOI:** 10.1186/s12964-020-00606-w

**Published:** 2020-08-31

**Authors:** Yameng Si, Jiadong Huang, Xiang Li, Yu Fu, Rongyao Xu, Yifei Du, Jie Cheng, Hongbing Jiang

**Affiliations:** 1 Jiangsu Key Laboratory of Oral Diseases, Nanjing Medical University, 136 Hanzhong Road, Nanjing, 210029 Jiangsu Province China; 2 The Affiliated Stomatological Hospital of Xuzhou Medical University, Xuzhou, China; 3 Department of Oral and Maxillofacial Surgery, Affiliated Hospital of Stomatology, Nanjing Medical University, Nanjing, China

**Keywords:** Venous malformation, AKT, FOXO1, Endothelial cells, Smooth muscle cells, TIE2-L914F

## Abstract

**Background:**

Venous malformations (VMs), most of which associated with activating mutations in the endothelial cells (ECs) tyrosine kinase receptor TIE2, are characterized by dilated and immature veins with scarce smooth muscle cells (SMCs) coverage. However, the underlying mechanism of interaction between ECs and SMCs responsible for VMs has not been fully understood.

**Methods:**

Here, we screened 5 patients with TIE2-L914F mutation who were diagnosed with VMs by SNP sequencing, and we compared the expression of platelet-derived growth factor beta (PDGFB) and α-SMA in TIE2 mutant veins and normal veins by immunohistochemistry. In vitro, we generated TIE2-L914F-expressing human umbilical vein endothelial cells (HUVECs) and performed BrdU, CCK-8, transwell and tube formation experiments on none-transfected and transfected ECs. Then we investigated the effects of rapamycin (RAPA) on cellular characteristics. Next we established a co-culture system and investigated the role of AKT/FOXO1/PDGFB in regulating cross-talking of mutant ECs and SMCs.

**Results:**

VMs with TIE2-L914F mutation showed lower expression of PDGFB and α-SMA than normal veins. TIE2 mutant ECs revealed enhanced cell viability and motility, and decreased tube formation, whereas these phenotypes could be reversed by rapamycin. Mechanically, RAPA ameliorated the physiological function of mutant ECs by inhibiting AKT-mTOR pathway, but also facilitated the nuclear location of FOXO1 and the expression of PDGFB in mutant ECs, and then improved paracrine interactions between ECs and SMCs. Moreover, TIE2 mutant ECs strongly accelerated the transition of SMCs from contractile phenotype to synthetic phenotype, whereas RAPA could prevent the phenotype transition of SMCs.

**Conclusions:**

Our data demonstrate a previously unknown mechanistic linkage of AKT-mTOR/FOXO1 pathway between mutant ECs and SMCs in modulating venous dysmorphogenesis, and AKT/FOXO1 axis might be a potential therapeutic target for the recovery of TIE2-mutation causing VMs.

**Video Abstract**

**Graphical abstract:**

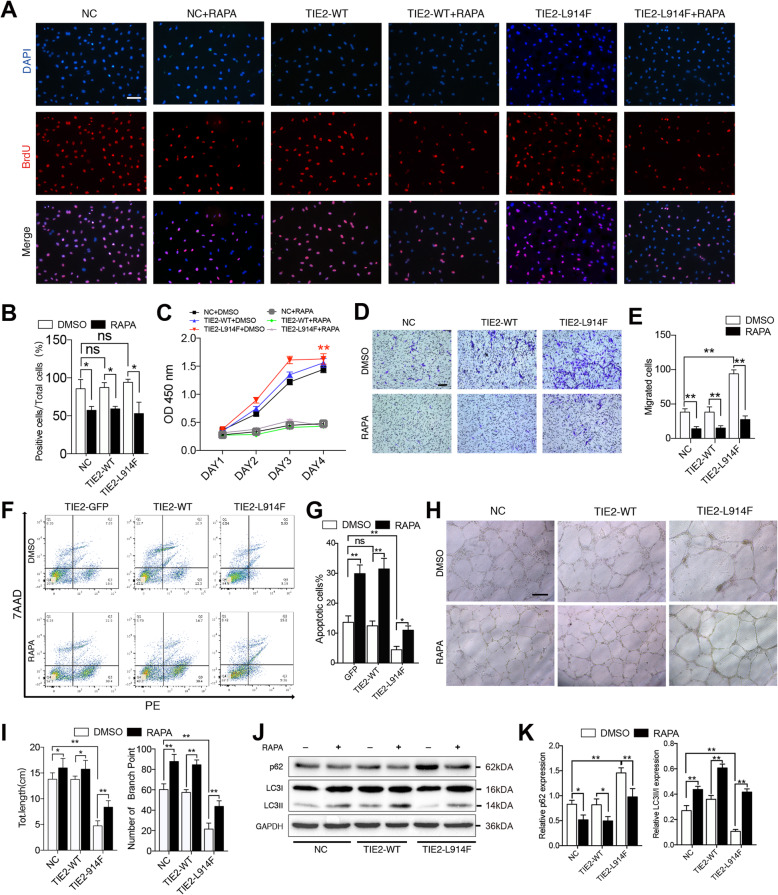

## Background

Venous malformations (VMs) are the most common vascular anomalies with an estimated incidence of about 1/10000 of the population. VMs result from an error in vascular morphogenesis and appear at birth and gradually expand with age, frequently occur in the oral and facial regions. Extensive VMs cause pain, bleeding, anatomic distortion and organ dysfunction [1–3]. Sclerotherapy is the traditional treatment, alone or in combination with surgical resection, but it has a series of side effects, such as skin necrosis, swelling and peripheral nerve deficits [2–6]. Current therapies are invasive and rarely curative as lesions tend to recur, so it needs to seek new therapies to alleviate clinical symptoms.

TIE2 (TEK), a member of the receptor tyrosine kinase subfamily, is mainly expressed in endothelial cells (ECs) [7]. TIE2 plays an important role in regulating angiogenesis, vascular remodeling, maturation, and integrity [8–10]. TIE2^−/−^ mice display impaired vascular branches and insufficient perivascular coverage [11]. Clinically, more than 95% of VMs are sporadic, and variable TIE2 mutations are detected in half of sporadic VMs [12, 13]. Somatic L914F (2740 C>T) as the most frequent change has been confirmed in a large proportion of sporadic VMs [14]. Remarkably, TIE2 autophosphorylation increase in all identified TIE2 mutant ECs [15–17]. In addition, human umbilical vein endothelial cells (HUVECs) engineered to express TIE2-L914F recapitulate VMs when injected in immunodeficient mice [18]. Therefore, an effective inhibitor of TIE2 signaling pathway tends to be a potential target for the treatment of VMs.

Rapamycin (RAPA), as a mammalian TOR (mTOR) signaling inhibitor, has been approved to use for preventing the rejection of transplanted organs and to block restenosis after angioplasty [19–21]. RAPA inhibits the kinase activity of mTORC1 when bound to FKBP12, whereas prolonged RAPA treatment inhibits mTORC2 assembly and AKT/PKB pathway [22–24]. RAPA, as an activator of autophagy, promotes angiogenesis during the recovery of heat-denatured ECs via AMPK/AKT/mTOR signaling [25], but RAPA has showed antiangiogenic activities in cancer linked to a decrease in production of vascular endothelial growth factor (VEGF) and a markedly inhibited response of ECs to stimulation by VEGF [26]. In addition, RAPA prevents TIE2-L914F mutant VMs growth in both murine model and human subjects by inhibiting p-AKT and increasing the number of α-SMA positive cells around the lumen [18]. However, the underlying mechanism is not entirely clear.

VMs are characterized by enlarged ECs-lined venous channels surrounded by sparse, irregularly distributed pericyte, and that is vascular smooth muscle cells (SMCs). Platelet-derived growth factor beta (PDGFB), as a secreted attractant from ECs, plays an essential role in pericyte recruitment. However, TIE2 mutants significantly hamper the ability of ECs to produce PDGFB [27]. As the target gene of FOXO1, the reduction of PDGFB depends on the phosphorylation of AKT [27]. Therefore, we hypothesize that RAPA might promote mutant ECs to produce PDGFB by inhibiting AKT/FOXO1 pathway, and then improve lumen integrity through reconciling the cross-talking between ECs and SMCs.

Here, we constructed TIE2-L914 mutant ECs and investigated the role of AKT/FOXO1/PDGFB in regulating cross-talking of mutant ECs and SMCs. TIE2 mutant ECs showed enhanced cell viability and motility, and a decreased tube formation, whereas these phenotypes could be reversed by RAPA. We also verified that FOXO1 was highly phosphorylated in TIE2 mutant ECs, and the secretion of PDGFB was reduced. Moreover, TIE2 mutant ECs strongly accelerated the transition of SMCs from contractile phenotype to synthetic phenotype. In contrast, RAPA could prevent the phenotype transition of SMCs. Our study reveals a previously unknown mechanistic linkage of AKT-mTOR/FOXO1 pathway between mutant ECs and SMCs in modulating venous dysmorphogenesis, suggesting it could be a potential therapeutic target for VMs.

## Methods

### Patients and samples

The current study was conducted on 20 formalin-fixed paraffin-embedded (FFPE) vascular anomalies tissues and 5 cases healthy control individuals (soft tissues from maxillofacial trauma patients) from Xuzhou Central Hospital during the period from 2017 to 2018. All cases were re-evaluated and confirmed by experienced professionals according to the current clinical, radiographic, and histopathological guidelines for VM.

Prior to this study, we obtained the informed consent of all patients and passed the review of the ethics committee.

### Mutation analysis

DNA was extracted from paraffin-embedded tissues of the VM patients by a QIAamp DNA FFPE Tissue Kit (Qiagen, Duesseldorf, Hilden, Germany). An approximately 300 bp DNA sequence of TEK 17 exon (NCBI Gene ID 7010) was amplificated by allele-specific PCR. The PCR primer sequences were as follows: TEK-F: 5′-TCTCTTAAATGTCATAGCTGTTCAG-3′, TEK-R: 5′-AGGGAACTCCACAGGAAAGAT-3′. TaKaRa Taq Version 2.0 plus dye (TaKaRa, Shiga, Tokyo, Japan) was used for PCR amplification. Subsequently, the product was examined by agarose gel electrophoresis, and the sequencing was performed by Sangon (Shanghai, China).

### Cell culture and treatment

Under a protocol approved by the Ethics and Research Committee of Nanjing Medical University, primary HUVECs and SMCs were obtained from fresh human umbilical vessels. Informed consent was obtained before volunteers were enrolled in this study. Primary HUVECs were cultured in medium dishes with ECM (Endothelial Cell Medium, Sciencell) containing 5% fetal bovine serum. SMCs were cultured in medium dishes with DMEM/F12 medium containing 10% fetal bovine serum. The medium was changed every 3 days until 80–90% confluence was achieved. To avoid genetic variation resulting from different individuals, all cells from three or more different donors were pooled together. Cells at no more than five passages were used for the following experiments. None-transfected and transfected ECs were treated with DMSO or rapamycin (15 nM) before every experiment.

### Lentiviral transfection

About 2 × 10^5^ HUVECs were seeded on six-well plate overnight than transfected with lentiviral vectors expressing the GFP, TIE-WT, and TIE2-L914F in the presence of polybrene (8 mg/ml). After incubation for 48 h, cells were treated with DMSO or rapamycin (MCE, Shanghai, China) for another 48 h for subsequent experiments.

### Apoptosis analysis

HUVECs (3 × 10^5^/well) were induced to undergo apoptosis via starvation in serum-free ECM medium overnight. After treated with DMSO or rapamycin for 48 h, cells were collected according to the protocol for fluorescein isothiocyanate (FITC) Annexin V Apoptosis Detection Kit (BioLegend, San Diego, CA, USA). Briefly, cells were resuspended in cold (4 °C) binding buffer and incubated for 15 min at room temperature following addition of 5 mL of Annexin V-FITC and 5 mL of 7-AAD solutions. Flow cytometry analysis was performed using FACSCalibur2 and analyzed with FlowJo 10.04 software. This experiment was independently repeated three times.

### Migration analysis

Migration assay was performed using a transwell chamber. Briefly, 8-μm pore size chambers with transparent polyester were placed into 24-well plates. 5 × 10^4^ cells of each group were seeded on up chamber, with cells starved overnight, complete ECM was placed on bottom chamber. After 24 h of incubation, non-migrated HUVECs were removed by cotton swabs. Migrated cells were fixed and stained with crystal violet (0.1%). Experiments were performed in duplicate, and the number of cells present in 5 fields per well was counted at × 10 magnification in a blinded manner using an EVOS microscope (Invitrogen).

### Bromodeoxyuridine (BrdU) assay

Before BrdU experiment, the cells had been transfected with lentivirus and pretreated with rapamycin for 48 h. 4 × 10^4^ ECs per well were seeded on 12-well plate. BrdU assay was performed as elsewhere described [28]. Briefly, after cells reacted with BrdU (0.03 mg/ml) for 24 h, the cell slides were fixed with 70% ethanol. Ten minutes later, the slides were washed three times (5 min each) using 0.1 M phosphate buffered saline (PBS). Then 1.5 M HCl (30 min) and 0.1 M boric acid (15 min) were used in turn. After blocking with normal goat serum for 1 h, BrdU antibody (Proteintech, #66241–1-Ig) was used at dilution of 1:200 overnight at 4 °C for immunofluorescence staining.

### CCK-8

After cells starved overnight, HUVECs (3000/well) were placed on 96-well plate with 100 μl complete ECM. Cells (at day 1, 2, 3 and 4 after plating) were added with CCK-8 10 μl per well. Four hundred fifty nanometer OD was tested after 3 h incubation.

### Tube formation assay

Tube formation assay was performed as elsewhere described [29, 30]. Briefly, 48 h following lentiviral transfection, HUVECs (2 × 10^4^ per well) were seeded in duplicate onto 96-well culture dishes coated with 50 μl of Matrigel (BD Biosciences). Tube formation was observed every 3 h post seeding with an inverted Olympus phase-contrast microscope, and five high-power fields at 100 magnifications were imaged randomly by using Olympus DP12 digital camera. The vessel length and branch points for each group were quantified by Angiogenesis Analyzer, a plug-in of ImageJ software. This experiment was independently repeated three times.

### Nucleoprotein separation and extraction

HUVECs were treated with rapamycin for an additional 48 h after they underwent serum-free starvation and then harvested. The nuclear proteins were isolated using the Invent Nuclear and Cytoplasmic Extraction Reagents Kit (Invent Biotechnologies, Plymouth, MN, USA) according to the manufacturer’s instructions.

### Western blot

The details of western blot were described elsewhere [31]. Briefly, cells were lysed by RIPA buffer (Beyotime, China), and the lysate was loaded onto 10% or 12% SDS-PAGE and then transferred to PVDF membranes (Millipore, USA). The membranes were blocked in 5% fat-free milk for 2 h before incubated with the following primary antibodies: GAPDH (Proteintech, #10494–1-AP), AKT (CST, #2920), p-AKT (CST, #4060), FOXO1 (CST, #2880), p-FOXO1 (CST, #9461), mTOR (CST, #2983), p-mTOR (CST, #5536), p62 (Proteintech, #18420–1-AP), LC3B (Abcam, #ab51520), α-SMA (Proteintech, #23081–1-AP), OPN (Proteintech, #22952–1-AP), Histone-H3 (Proteintech, #17168–1-AP) at 4 °C overnight. Then the membranes were incubated for 30 min at 37 °C with secondary antibodies after washing. ImageJ software was used for quantitatively analysis.

### Real-time quantitative PCR

Total RNA from cultured cells was extracted using Trizol reagent (Vazyme, Nanjing, Jiangsu, China) according to the manufacturer’s instructions. Quantitative real-time PCR analyses were performed in triplicate using SYBR Green PCR Master Mix (TaKaRa), and reactions were detected using an Applied Biosystems 7300 Real-time PCR system (Applied Biosystems, Gaithersburg, CA, USA). The primer sequences used for real-time PCR are listed as follows. PDGFB-F: 5′-TCTCTGCTGCTACCTGCGTCTG-3′, PDGFB-R: 5′-AGGTCCAACTCGGCTCTGTCTTC-3′; TIE2-F: 5′-GGGACTTTGCAGGAGAACTGG-3′, TIE2-R: 5′-AAATGCTGGGTCCGTCTCCA-3′.

### Immunostaining

Immunohistochemical staining was performed using continuous paraffin sections of venous malformations. Details of immunohistochemical staining are described elsewhere [31]. Briefly, the serial tissue sections were immersed in 3% H_2_O_2_ for 30 min. Then, the sections were washed (3 × 5 min) in 0.1 M phosphate buffered saline (PBS). Afterwards, the sections were incubated with normal goat serum for 1 h at room temperature. PDGFB (Abclonal, #A1195) and α-SMA (Proteintech, #23081–1-AP) were used as primary antibodies to detect relative molecular expression. The sections were treated with primary antibodies at 4 °C overnight and then visualized by diaminobenzidine (DAB) kit after incubating with second antibody for 40 min. Finally, the sections were counterstained with hematoxylin.

### Immunofluorescence

NC, TIE2-WT, TIE-L914F HUVECs 5000/well were seeded on 12-well plate coating with slides. Immunofluorescence staining was performed after cells were treated with DMSO or rapamycin for 48 h. Briefly, cell slides were fixed with 4% paraformaldehyde for 15 min and then washed with 0.1 M Phosphate buffered saline (PBS) three times. After 10 min of drilling with 0.1% Triton X-100 (Beyotime, Shanghai, China), the sections were treated with normal goat serum for 1 h at 37 °C, and then anti-mouse FOXO1 was used as primary antibody at dilutions of 1:200 overnight at 4 °C.

### Elisa

The concentration of PDGFB in the supernatants of the cultured HUVECs was detected by ELISA kits (Human PDGFB; CUSABIO, Wuhan, China) according to the manufacturer’s procedures.

### Co-culture systems

We established the coculture system through 6-well plate and 3 μm pore size transwell inserts (Corning). The SMCs (5 × 10^5^ per well) were seeded in 6-well plate and the same quantity of different treated ECs were seeded in the transwell inserts located in adjoining wells. The transwell inserts with ECs were moved to the wells containing SMCs when cells attached to the wall firmly (24 h). After another 48-h culture, SMCs were harvest for western blot. Similarly, we set up another co-culture system for migration experiments, 24-well plate and 8 μm pore size transwell chambers (Corning) were used instead, 5 × 10^4^ different treated ECs per well were seeded in 24-well plate and the same number of SMCs were seeded in up chambers adjacently. After cells attached firmly, the transwell chambers with ECs were moved to the wells containing ECs. Forty eight hours later, chambers were taken out for migration experiments.

### Statistical analysis

The results are represented as means ± SEM or as specified in the figure legends. The statistical analysis was performed using Prism 7 software (GraphPad Software, La Jolla, CA, USA). Comparisons between the 2 groups were analyzed using the 2-tailed, unpaired Student’s t test. Comparisons among ≥3 groups were performed using 1-way ANOVA followed by Tukey’s multiple comparisons. Adjusted values of *P*<0.05 were considered significant.

## Results

### Reduced PDGFB and α-SMA expression in VMs with TIE2-L914F mutation

**Fig. 1:**
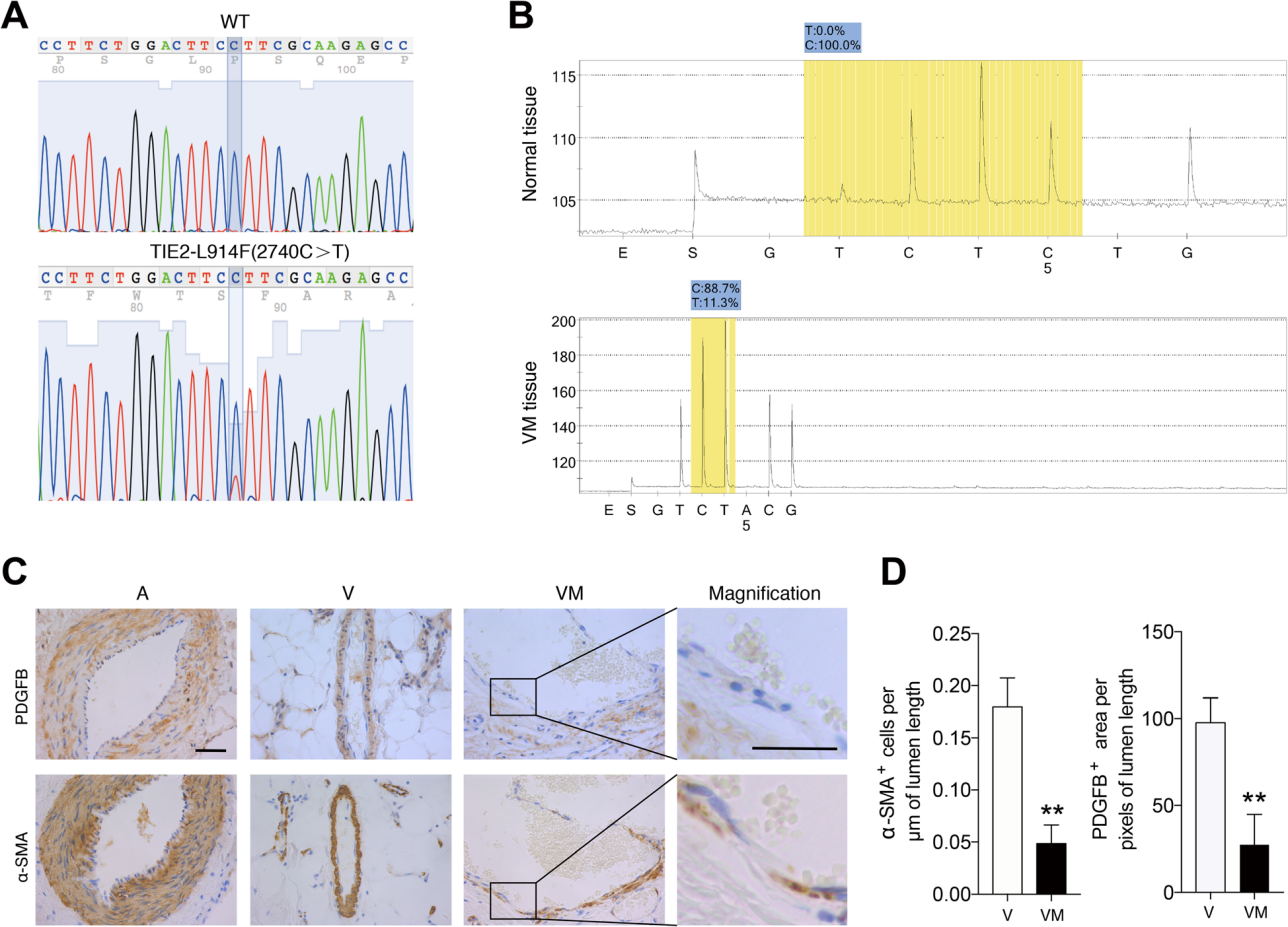
Reduced PDGFB and α-SMA expression in patients with TIE2-L914F mutant venous malformation. **a** Representative DNA sequences of VM patients (below) and healthy controls (up) detected by SNP site sequencing. **b** Mutation rate of TIE2-L914F in VM patients and healthy controls detected by pyrosequencing. **c** Representative immunohistochemical images of arteries (A), normal veins (V), VM veins (VM). Immunohistochemistry staining images showing expression levels of PDGFB (up) and α-SAM (below) in A, V and VM. On the far right are the magnifications of the VM images. **d** α-SAM positive cells per μm of lumen length and PDGFB positive area per pixels of lumen length in the V and VM groups were calculated. Scale bar, 50 μm. The data are means ± SEM (*n* = 6). **P < 0.01

We extracted DNA from paraffin section specimens from 20 patients diagnosed as VMs and found that 5 of them were identified as TIE2-L914F mutation (2740 C>T) (Fig. 1a and Additional file 1: Figure S1). Pyrosequencing revealed the mutation rate at this site is approximately 11% (Fig. 1b). Subsequently, paraffin sections of these patients with TIE2-L914F mutation were undergone HE stain, compared to normal vessels, malformed vessels have larger and thinner lumens (see Additional file 1: Figure S2). To investigate the regulatory relationship between ECs and pericytes, the expression of PDGFB was detected by immunohistochemistry. As a result, the expression of PDGFB around the lumens was significantly decreased. Meanwhile, the number of α-SMA positive cells around the lumen decreased conformably (Fig. 1c, d). These data suggest that VMs with TIE2-L914F mutation have low expression of PDGFB, which may be one of the reasons for the insufficient coverage of smooth muscle cells in malformed veins.

### TIE2-L914F mutation activates AKT/FOXO1 signaling

**Fig. 2:**
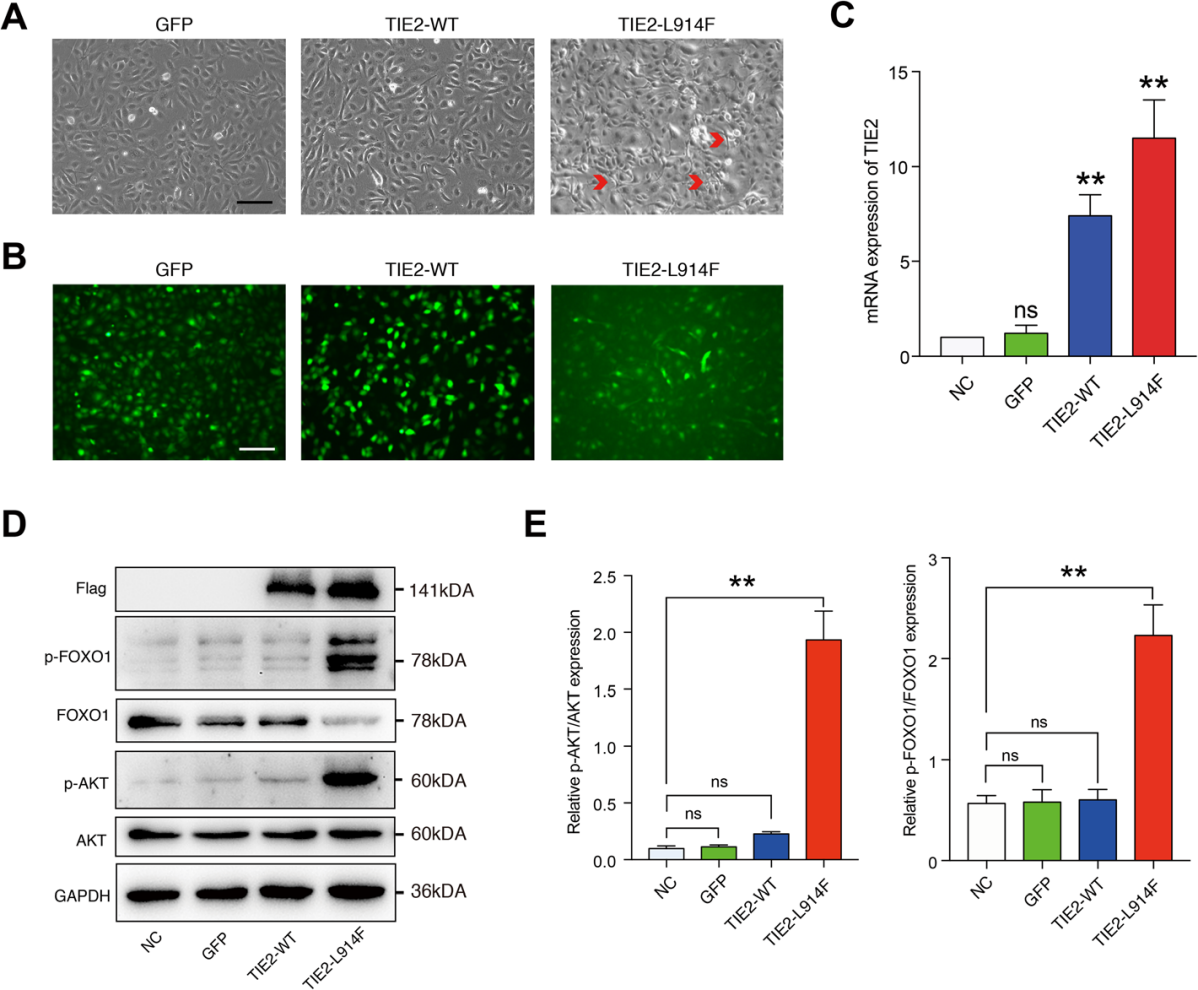
TIE2-L914F mutation activates AKT/FOXO1 signaling. **a** ECs were transfected with lentivirus expressing GFP, TIE2-WT, TIE2-L914F respectively. Red arrows: typical elongated cell morphology of ECs. **b** ECs expressing lentivirus vectors under fluorescence microscopy. **c** Relative mRNA level of TIE-2. **d, e** 48 h after transfecting, the expression levels of AKT, p-AKT (ser473), FOXO1 and p-FOXO1 in ECs were then measured by Western blot. Flag tags were used to verify effectiveness of transfection. Scale bar, 200 μm. The data are means ± SEM (*n* = 3). ns: no significant, **P < 0.01

ECs from the umbilical vein of five different newborns were collected and mixed for the following experiments. ECs showed a typical cuboidal cobblestone morphology (see Additional file 1: Figure S3A) and were identified as endothelial cells by immunofluorescent staining with vWF primary antibody (see Additional file 1: Figure S3B). To investigate the biological behavior of TIE2-L914F mutant ECs, we constructed lentivirus vectors expressing TIE2-L914F to transfect ECs. The mutant sequence was showed in Additional file 2. TIE2-L914F mutant ECs showed highly overlapping and elongated cell morphology, obviously distinct from the typical cuboidal cobblestone morphology characteristics of NC (none-transfect), GFP and TIE2-WT (Fig. 2a). The perturbed monolayer is not due to chronic activation of normal TIE2 signaling but represents a mutation-specific effect [32]. We observed the target genes were transfected effectively through fluorescence microscope and real-time quantitative PCR assay (Fig. 2b, c). As expected, TIE2 mRNA level of both TIE-WT and TIE2-L914F increased after lentivirus transfection (Fig. 2c). Expression of flag tag protein also confirmed the success of transfection (Fig. 2d). However, only TIE2-L914F mutant ECs showed abnormal phosphorylation of AKT (ser 473) which is downstream of TIE2. FOXO1, a downstream molecular signaling pathway of AKT, was also significantly phosphorylated (Fig. 2d, e). These results suggest that ECs with specific site mutation (TIE2-L914F mutation) are morphologically altered and abnormally activate the AKT/FOXO1 signaling pathway.

### Effects of TIE2-L914F mutation on the biological characteristics of ECs

**Fig. 3:**
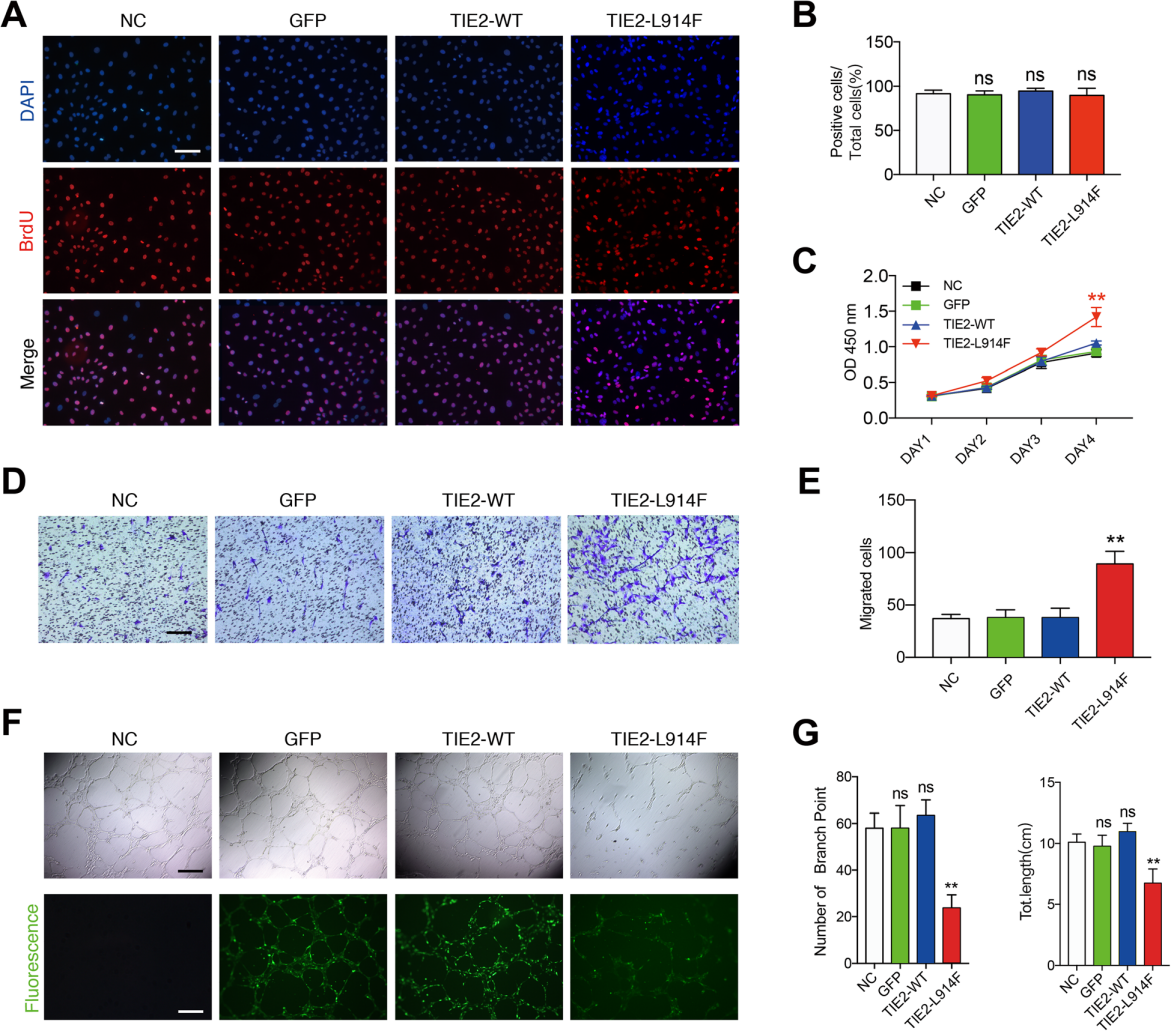
Effects of TIE2-L914F mutation on the biological characteristics of ECs. **a, b** 48 h after transfecting, ECs underwent BrdU assay. The ratio of positive cells to total cells in each visual field was analyzed by ImageJ software. **c** Different treated ECs underwent CCK-8 experiment, the OD values of 450 nm at day 1, 2, 3, 4 were tested. **P < 0.01 TIE-L914F versus NC, GFP and TIE2-WT. **d, e** Transwell assay was used to study the migration ability of NC-ECs, GFP-ECs, TIE2-WT-ECs and TIE2-L914F-ECs over a period of 24 h. **f** Tube formation assay of different groups of ECs. (up: inverted microscope; below: fluorescence microscope). **g** Branch points (left) and total length (right) of each group were measured. Scale bars, 100 μm (**a**), 200 μm (**d**), 500 μm (**f**). The data are means ± SEM (*n* = 3). ns: no significant, **P < 0.01

To explore the biological characteristics of TIE2-L914F mutant ECs, we performed BrdU and CCK-8 assay. No significant difference in cell proliferation can be detected among all groups (Fig. 3a, b), but the activity of TIE2-L914F mutant ECs was significantly increased compared with the other three groups (Fig. 3c). We speculate that this might be related to the enhanced anti-apoptotic ability associated with the phosphorylation of AKT. Next, we examined the migration capacity of different treated ECs and found that TIE2-L914F mutant ECs showed a stronger migration capacity than other groups (Fig. 3d, e). Subsequently, tube formation assay was performed. We selected the 12th hour as the optimal observation time, when the tube-like structures did not begin to fade in both normal ECs and mutant ECs. An impaired tube formation ability was demonstrated in TIE2-L914F mutant ECs (Fig. 3f, g). Taking together, TIE2-L914F mutant ECs have increased cell viability and motility, and impaired tube formation, which might be responsible for the formation of venous malformations.

### AKT/FOXO1 pathway inhibition amends the abnormal characteristics of TIE2-L914F mutant ECs

**Fig. 4:**
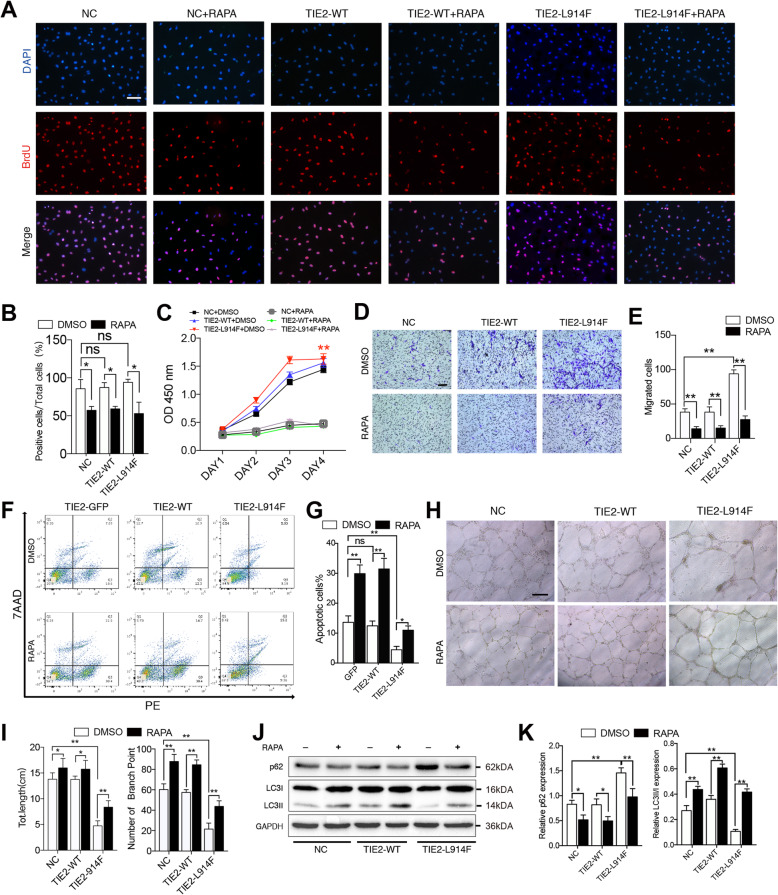
AKT/FOXO1 pathway inhibition amends the abnormal characteristics of TIE2-L914F mutant ECs. **a, b** BrdU assay was performed to study the proliferation of NC-ECs, TIE2-WT-ECs and TIE2-L914F-ECs treated with DMSO or rapamycin. **c** CCK-8 experiment was performed to study the effects of rapamycin on the cell activity of NC-ECs, TIE2-WT-ECs and TIE2-L914F-ECs. **P < 0.01 TIE-L914F versus TIE2-L914F + RAPA **d, e** Transwell assay was used to study the effects of rapamycin on the migration ability of NC-ECs, TIE2-WT-ECs and TIE2-L914F-ECs over a period of 24 h. **f, g** Apoptotic assay showed the apoptosis of NC-ECs, TIE2-WT-ECs and TIE2-L914F-ECs, and the effect of rapamycin on cellular apoptosis. **h, i** Tube formation assay was carried on NC-ECs, TIE2-WT-ECs and TIE2-L914F-ECs treated with DMSO of rapamycin. The total length and the number of branch points were used as indicators. **j, k** Expression of autophagy related indexes (p62, LC3I, LC3II) in different treated ECs was detected by western blot. Scale bars, 100 μm (**a, d**), 500 μm (**h**). The data are means ± SEM (*n* = 3). ns: no significant, *P < 0.05, **P < 0.01

To further verify the dysregulated roles of AKT/FOXO1 pathway in TIE2-L914F mutant ECs, RAPA was used to inhibit AKT/FOXO1 pathway. Both BrdU and CCK-8 assay showed that RAPA significantly inhibited the proliferation of ECs (Fig. 4a, b, c). Whereas, RAPA could reduce the number of migrating ECs whether non-transfected or transfected groups (Fig. 4d, e). TIE2-L914F mutant ECs showed lower apoptosis rate than the group of GFP or TIE2-WT, suggesting their enhanced anti-apoptotic ability, consistent with the above speculation. Meanwhile, RAPA significantly upregulated the proportion of early apoptosis cells (Fig. 4f, g). Tube formation assay revealed that RAPA increased the total length of tubes and the number of branch nodes in each group. Even more remarkably, RAPA improved the tube-forming ability of TIE2-L914F mutant ECs (Fig. 4h, i). RAPA has been reported to promote autophagy [33], so we examined the expression of autophagy related indicators after cells starving overnight, and found that p62 expression increased and the ratio of LC3II/I decreased in TIE2-L914F mutant ECs. However, when RAPA was added for 48 h, it turned out to be an autophagy accelerant of TIE2-L914F mutant ECs, showing an increased p62 and decreased ratio of LC3II/I (Fig. 4j, k). These results suggest that RAPA inhibits the proliferation and migration of ECs and restores the apoptosis of TIE2-L914F mutant ECs nearly to a normal level, thereby, improves lumen maturity of TIE2-L914F mutant ECs by promoting autophagy.

### RAPA promotes nuclear location of FOXO1 and PDGFB expression

**Fig. 5:**
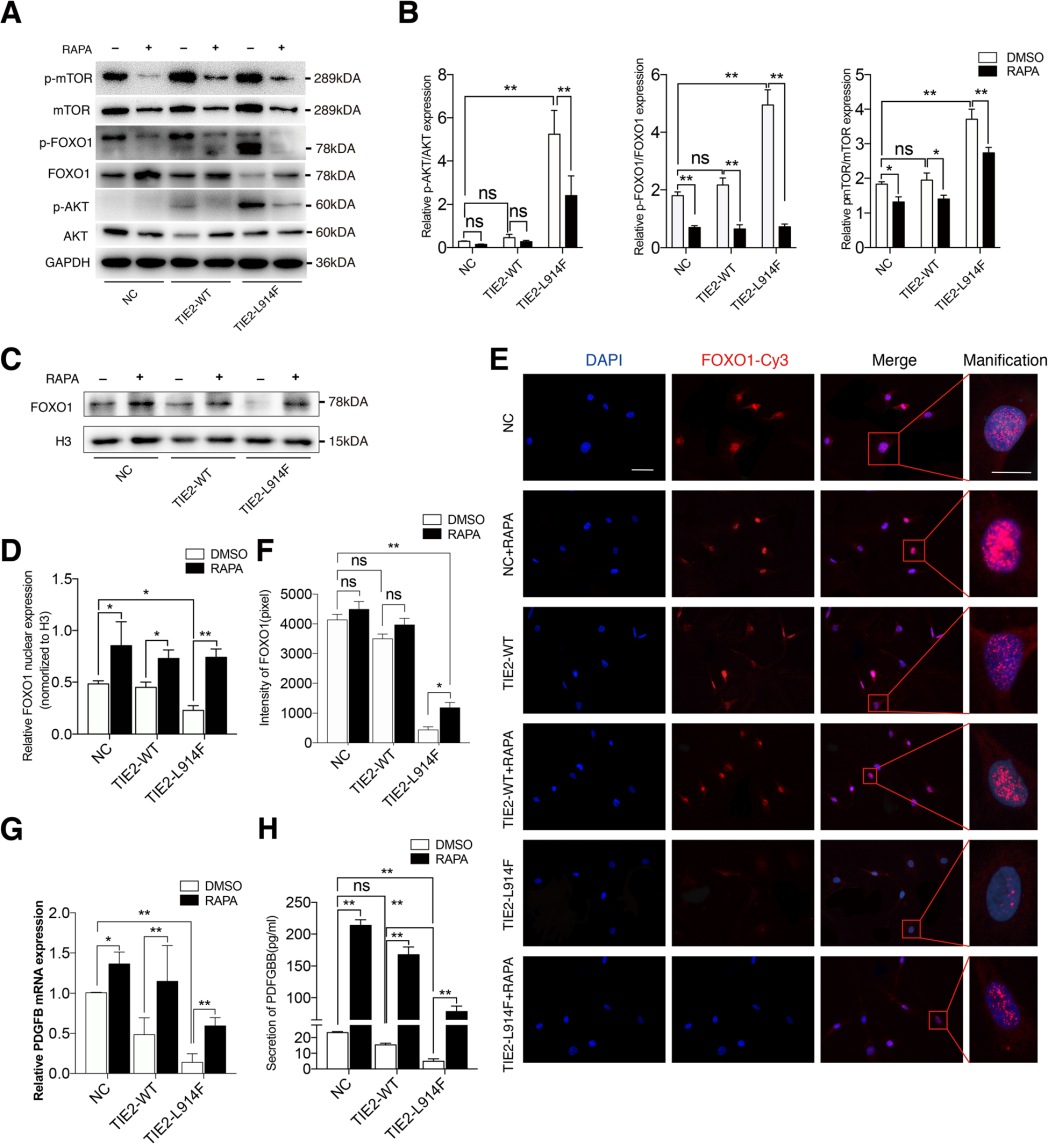
RAPA promotes nuclear location of FOXO1 and PDGFB expression. **a, b** Western blot analysis of AKT, p-AKT, FOXO1, p-FOXO1, mTOR and p-mTOR on different groups of ECs treated with DMSO or rapamycin. **c, d** Expression of FOXO1 in the nucleus was detected by western blot. **e** NC-ECs, TIE2-WT-ECs and TIE2-L914F-ECs were subjected to IF staining with anti-FOXO1 antibody after the RAPA (15 nM) treatments for 48 h. **f** Average fluorescence intensity of each field was statistically analyzed by ImageJ software. **g** Expression levels of PDGFB were measured using real-time quantitative PCR. **h** Production of PDGFB released from the ECs was determined using ELISA. Scale bar, 50 μm. The data are means ± SEM (*n* = 3). ns: no significant, *P < 0.05, **P < 0.01

A mature blood vessel is maintained by pericytes and ECs, the decreased expression of PFGFB in patients with TIE2-L914F mutation indicates the impaired recruitment of pericytes. Although previous results have shown that RAPA acts on ECs to improve immature blood vessels, whether RAPA affects the recruitment of pericytes through AKT/FOXO1 pathway remains unknown. Our results showed that RAPA down-regulated the phosphorylation of AKT, FOXO1, and mTOR, especially in the TIE2-L914F mutant group (Fig. 5a, b). FOXO1 is a transcription factor that functioned in nucleus, so we extracted nuclear proteins and analyzed FOXO1 expression in nucleus, and found that FOXO1 expression in nucleus significantly reduced in TIE2-L914F mutant ECs, whereas there was an increased expression of FOXO1 after the addition of RAPA (Fig. 5c, d). Subsequently, we performed immunofluorescence staining to detect nuclear location of FOXO1 and further verified that RAPA promoted nuclear location of FOXO1 (Fig. 5e, f). To detect the effect of RAPA on ECs, PDGFB, as a FOXO1 target gene, was examined, and the results showed that RAPA promoted PDGFB expression, but also reversed the decreased expression of PDGFB caused by TIE2-L914F mutation (Fig. 5g). As PDGFB function as an identity of secretory protein, we detected the expression of its subtype PDGFBB in the supernatant of cell culture, and the result of ELISA assay is consistent with that obtained by real-time quantitative PCR (Fig. 5h). Therefore, RAPA effectively inhibits the phosphorylation of FOXO1 and facilitates its entry into the nucleus, in turn activates the transcription of PDGFB gene, which is related to the recruitment of pericytes.

### RAPA promotes the interaction of ECs and SMCs

**Fig. 6:**
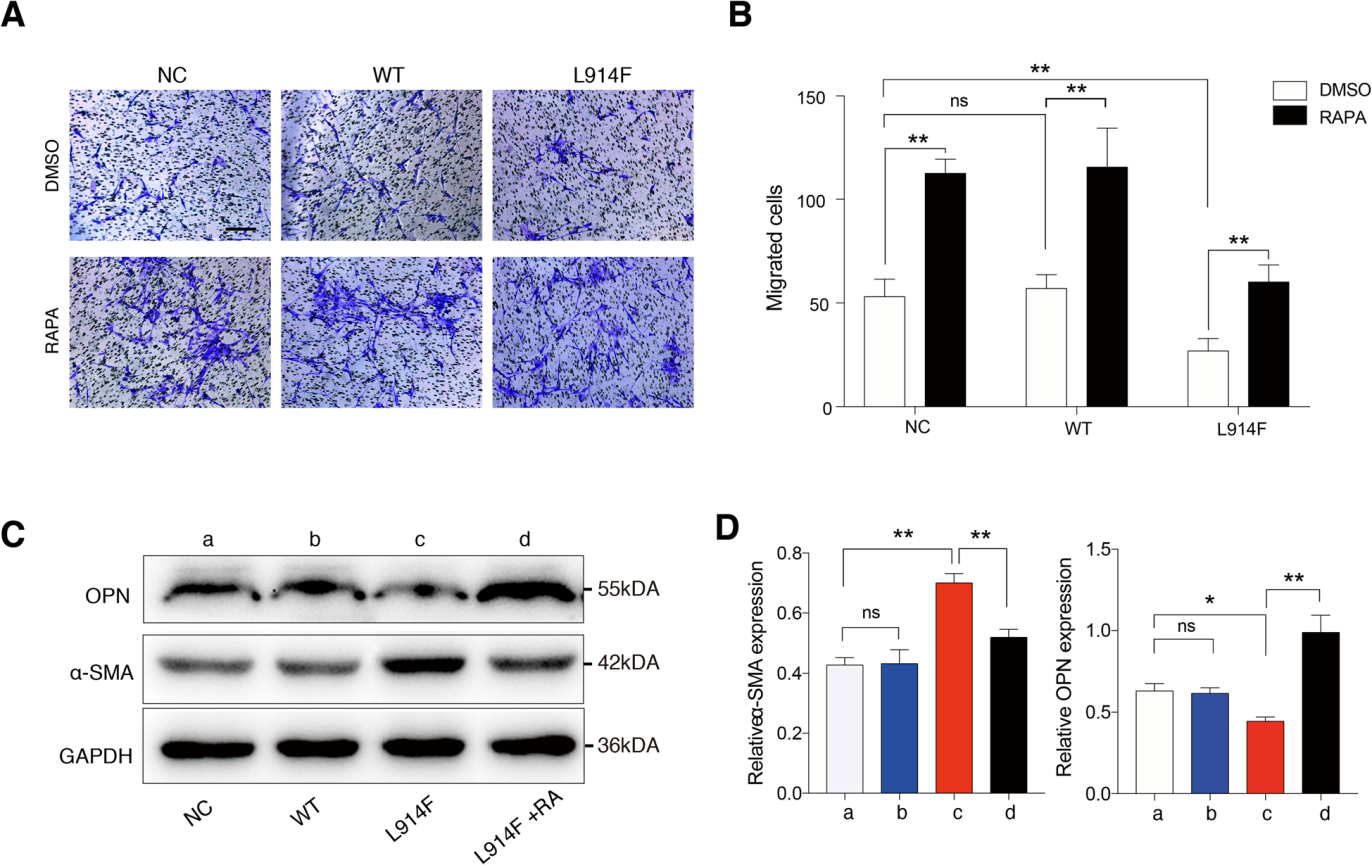
RAPA promotes the interaction of ECs and SMCs. **a, b** Transwell assay was used to study the migration ability of SMCs after co-culturing with NC, TIE2-WT and TIE2-L914F mutant ECs treated with DMSO or rapamycin over a period of 24 h. **c, d** Different groups of ECs were treated with DMSO or rapamycin (RAPA), and then co-cultured with SMCs. The expression of OPN and α-SMA were measured in SMCs by western blot. Scale bar, 100 μm. The data are means ± SEM (*n* = 3). ns: no significant, *P < 0.05, **P < 0.01

To further investigate whether RAPA could improve effects of TIE2-L914F mutant ECs on SMCs, we co-cultured two kinds of cells using a transwell chamber. Compared to NC and WT groups, TIE2 mutation group showed a decreased migration of SMCs, whereas no significant differentiation between NC and WT (Fig. 6a, b). Moreover, the migration capability of SMCs was all enhanced in 3 groups after ECs treated with rapamycin for 48 h (Fig. 6a, b). Phenotype associated proteins of SMCs were also examined after co-culture. A reduced osteopotin (OPN, a synthetic-associated protein) and enhanced α-SMA (a contractile-associated protein) were significantly showed in SMCs co-cultured with TIE2-L914F mutant ECs. Importantly, this phenotype of SMCs could be reversed by RAPA. (Fig. 6c, d). These results suggest that TIE2-L914F mutant ECs accelerate the transition of SMCs from synthetic phenotype to contractile phenotype, and RAPA can reverse this transition.

## Discussion

VMs are characterized by dilated and immature veins with scarce SMCs coverage. Here, we reveal a mechanistic linkage of AKT-mTOR/FOXO1 pathway between mutant ECs and SMCs in modulating venous dysmorphogenesis. RAPA, functioned as an inhibitor of AKT/FOXO1/PDGFB and AKT/mTOR signaling pathway, shows a potential therapeutic effect in TIE2 mutant VMs.

Previous studies have found that somatic mutations of TIE2 exist in about 50% of VMs [13], in the other 50%, about 27% have mutations of PIK3CA, downstream of TIE2 [34]. TIE2-L914F mutation accounts for the majority (about 77%) of all sporadic VMs [17]. In this study, we included 20 patients who were pathologically diagnosed with VMs. 5 of them were detected having TIE2-L914F mutation (Fig. 1a and Additional file 1: Figure S1). The 25% mutation rate was slightly lower than that reported in the literature, which may be due to ethnic difference.

The TIE2 receptor activates the PI3K/AKT pathway by binding its ligands Ang1 and Ang2 [35, 36]. TIE2-L914F mutation in ECs can cause TIE2 ligand independent autophosphorylation and enhanced AKT signaling [37]. FOXO1, a member of the forkhead family, is the main signaling molecule downstream of the PI3K/AKT pathway. When PI3K activates the phosphorylation of AKT and FOXO1, p-FOXO1 is restricted in the cytoplasm, and loses its transcription factor activity [38, 39]. Furthermore, published evidence indicated that FOXO1 null mice exhibit vascular defects [40], suggesting that FOXO1 plays an important role in the vasculature. In our study, the expression levels of p-AKT, p-FOXO1 in TIE2-L914F mutant ECs were significantly up-regulated, and the migration and anti-apoptotic ability of TIE2 mutant ECs were enhanced, but TIE2 mutant ECs showed impairment of forming integrated and mature tubular structures. These results may account for the increased number but irregular morphology of the vessels in VMs and its anti-apoptotic ability may explain the reasons that malformed veins do not subside despite insufficient wall cell support.

RAPA is used to treat patients with serious VMs and achieved better results than TIE2-TKI (the inhibitor of TIE2) [18]. This is because RAPA could inhibit AKT signaling bypass TIE2. Previous studies have reported that RAPA could promote tube formation during the recovery of heat-denatured ECs via AMPK/AKT/mTOR signaling [24, 25], suggesting mTOR-mediated autophagy may be involved in vascular remodeling. However, whether autophagy plays a role in TIE2 mutant VMs has not been reported. In this study, we first found that RAPA restored the changes of cell characteristics caused by L914F mutation, including migration, apoptosis and tube formation. The results of western blot shown a reduction in autophagy of TIE2-L914F mutant ECs and RAPA could improve angiogenesis by promoting autophagy in mutant ECs. RAPA first inhibits the kinase activity of mTORC1, yet prolonged RAPA treatment inhibits mTORC2 (the one that directly phosphorylates AKT at S473) [24]. Since abnormal phosphorylation of the AKT pathway is prevalent in somatic mutant VMs [41], we detected the expression of AKT and downstream FOXO1 after RAPA treatment and found it inhibited the AKT/FOXO1 pathway, leading to intracellular aggregation of FOXO1.

Recently gene microarray analysis showed that increased phosphorylation of FOXO1 can down-regulate the genes related to vascular development and remodeling, including Ang2, BMP4, PDGFB [27]. PDGFB is a strong mitogen and chemoattractant of mesenchymal cells, and the lack of PDGFB or PDGFR-β in mice will lead to insufficient coverage of pericytes, which leads to vascular dilation and endothelial hyperplasia [42]. ECs from the growing vessels secrete PDGFB which interacts with PDGFR-β on pericytes and triggers their recruitment to stabilize the nude capillary tube. The signaling network of PDGFB and PDGFR-β exerts a vital role in the pericyte recruitment to the vessels that are being newly formed [43, 44]. Our results indicate a decrease in PDFGB expression and insufficient smooth muscle cell coverage in paraffin sections of patients diagnosed with TIE2-L914F mutant VMs. In addition, the transcriptome expression of PDGFB was up-regulated and the secretion of PDGFB was increased in RAPA-treated mutant ECs. The decrease of PDGFB is one of the important reasons for the insufficient coverage of perivascular wall cells in venous malformations. So, efficacy of RAPA in treating VMs lies in activating FOXO1, thereby indirectly promoting the secretion of PDGFB.

The cross-talking between ECs and SMCs is a key linkage during vascular maturation. ECs secrete chemokines to recruit SMCs/pericytes, which migrate and proliferate around the endothelial cell tube, participating in remodeling the endothelial cell tube into a mature vascular structure [45–47]. Dysfunction of SMCs can lead to a variety of vascular diseases. Normal, mature and stable SMCs contain abundant muscle fibers, with poor proliferation and migration ability, which are contractile phenotype. In response to vascular injury, the activated inflammatory cells, platelets and SMCs release the growth factors, especially platelet-derived growth factor (PDGF), thereby leading to a switch of SMCs from a contractile phenotype to a synthetic phenotype [48–50]. PDGFB is described to be one of the most potent stimulants for SMCs proliferation and migration [51]. In early vascular development, synthetic phenotypic SMCs are required to migrate around lumens, proliferate and secrete extracellular matrix to promote intercellular connections. Osteopontin (OPN), a multifunctional protein which highly expressed in synthetic SMCs, is often used as a marker for synthetic SMCs [52]. α-SMA, a contractile-associated protein [53], was also tested in this study. The results showed TIE2-L914F mutant ECs not only prevented the migration of SMCs, but also inhibited the expression of OPN and promoted the expression of α-SMA in SMCs. This suggests that SMCs transform from synthetic phenotype to contractile phenotype. RAPA could reverse these phenotypes of SMCs. These findings link TIE2-L914F mutant ECs and SMCs phenotype to form VMs through AKT/FOXO1/PDGFB pathway. Application of inhibitor of the AKT pathway may provide a potential therapeutic strategy for TIE2-L914 mutant VMs.

## Conclusions

Our study revealed that RAPA maintained the vascular lumen integrated by improving the biological properties of TIE2-L914F mutant ECs. Meanwhile, RAPA improved the low autophagy condition induced by TIE2 mutation. On the other hand, PDGFB, secreted by ECs, was increased by inhibiting AKT pathway, which changed the phenotype and may accelerate the recruitment of SMCs. These findings suggested the inhibitor of AKT pathway may be a potential target for the treatment of refractory venous malformations.

## Supplementary information

**Additional file 1: Figure S1.** SNP sequencing results of four other patients diagnosed with TIE2-L914 caused VMs. The red arrows indicate the mutation site. **Figure S2.** A representative HE stained section of a patient with venous malformation. The dotted line indicates a malformed vein. VM: venous malformation. Scale bar, 200 μm. **Figure S3.** (A) A representative picture of ECs morphology. (B) Immunofluorescence showed cells extracted from umbilical cord expressing vWF. Scale bar, 200 μm.

**Additional file 2.** The mutant sequence of TIE2-L914F.

## Data Availability

All data generated or analysed during this study are included in this published article and its supplementary information files.

## Abbreviations

VMs: Venous malformations
ECs: Endothelial cells
SMCs: Smooth muscle cells
PDGFB: Platelet-derived growth factor beta
HUVECs: Human umbilical vein endothelial cells
TIE2: Receptor tyrosine kinase with immunoglobulin and epidermal growth factor homology domains-2
ELISA: Enzyme-linked immunosorbent assay
CCK-8: Cell counting kit-8
WT: Wild type
RAPA: Rapamycin
AKT: Protein Kinase B
α-SMA: α-smooth muscle actin
OPN: Osteopontin
IF: Immunofluorenscence
GFP: Green fluorescent protein
TEK: Tyrosine kinase, endothelial
vWF: Factor VII related antigen
ECM: Endothelial cell medium
DMSO: Dimethyl sulfoxide
FOXO1: Factors transcription forkhead 1
