# Supplementary figures and images for: AKT/FOXO1 axis links cross-talking of endothelial cell and pericyte in TIE2-mutated venous malformations
Source: Cell Commun Signal. 2020 Aug 31;18:139. doi: 10.1186/s12964-020-00606-w (PMC7457504; doi:10.1186/s12964-020-00606-w)

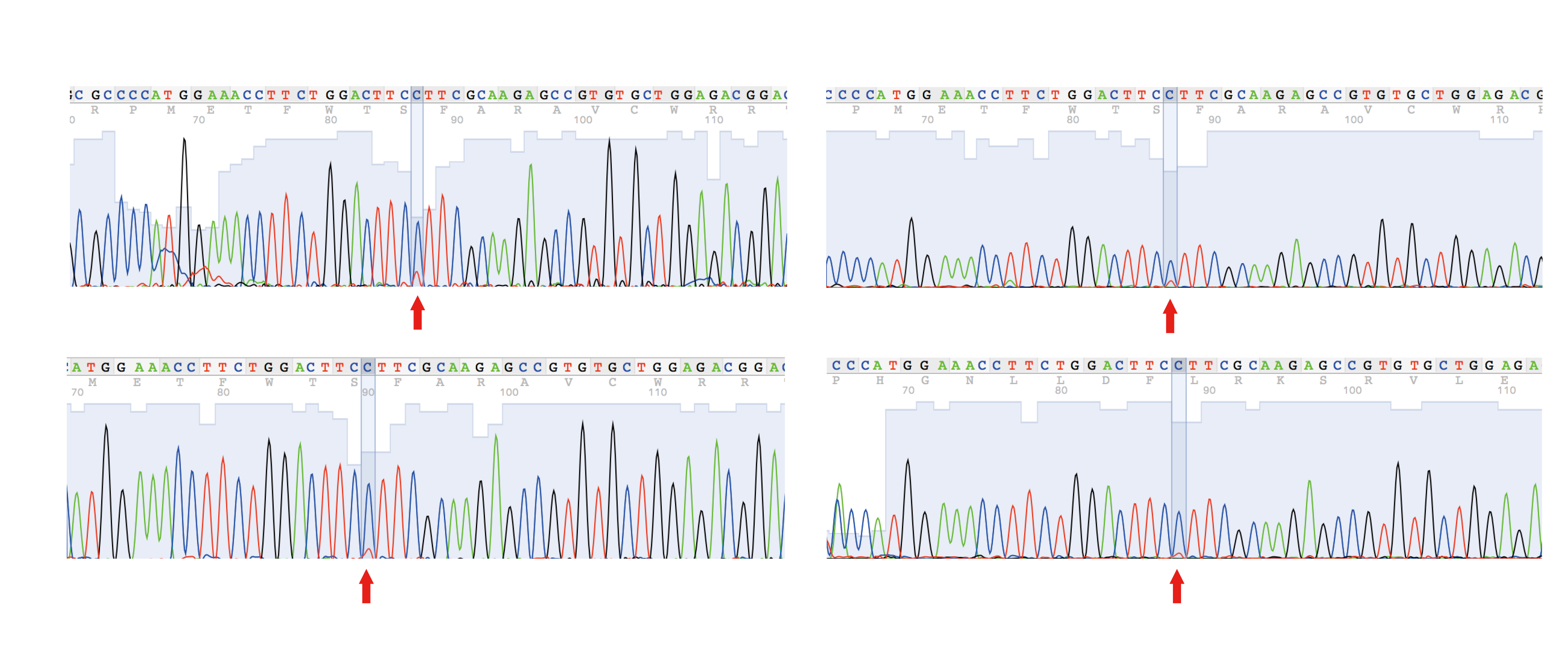

Supplement: Supplementary file 2 — Additional file 1: Figure S1. SNP sequencing results of four other patients diagnosed with TIE2-L914 caused VMs. The red arrows indicate the mutation site. Figure S2. A representative HE stained section of a patient with venous malformation. The dotted line indicates a malformed vein. VM: venous malformation. Scale bar, 200 μm. Figure S3. (A) A representative picture of ECs morphology. (B) Immunofluorescence showed cells extracted from umbilical cord expressing vWF. Scale bar, 200 μm. [file 12964_2020_606_MOESM2_ESM.zip › Figure S1.tif]

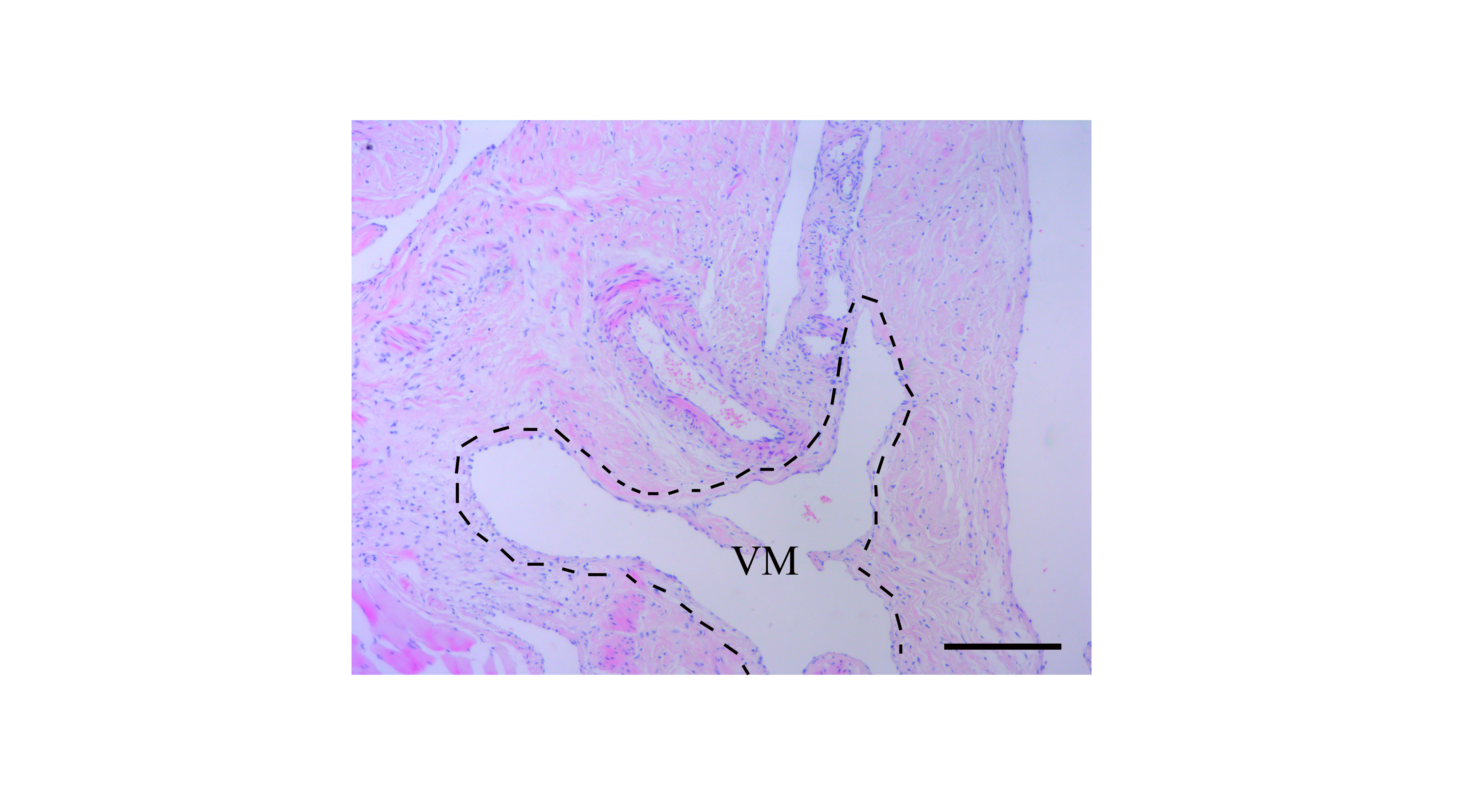

Supplement: Supplementary file 2 — Additional file 1: Figure S1. SNP sequencing results of four other patients diagnosed with TIE2-L914 caused VMs. The red arrows indicate the mutation site. Figure S2. A representative HE stained section of a patient with venous malformation. The dotted line indicates a malformed vein. VM: venous malformation. Scale bar, 200 μm. Figure S3. (A) A representative picture of ECs morphology. (B) Immunofluorescence showed cells extracted from umbilical cord expressing vWF. Scale bar, 200 μm. [file 12964_2020_606_MOESM2_ESM.zip › Figure S2.tif]

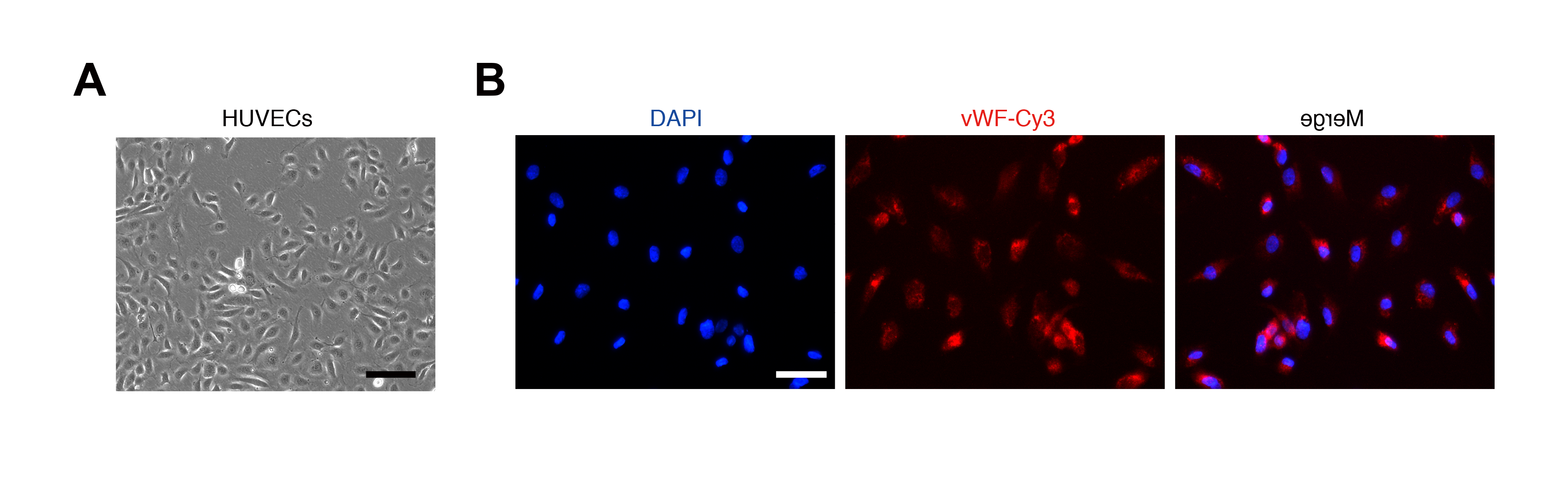

Supplement: Supplementary file 2 — Additional file 1: Figure S1. SNP sequencing results of four other patients diagnosed with TIE2-L914 caused VMs. The red arrows indicate the mutation site. Figure S2. A representative HE stained section of a patient with venous malformation. The dotted line indicates a malformed vein. VM: venous malformation. Scale bar, 200 μm. Figure S3. (A) A representative picture of ECs morphology. (B) Immunofluorescence showed cells extracted from umbilical cord expressing vWF. Scale bar, 200 μm. [file 12964_2020_606_MOESM2_ESM.zip › Figure S3.tif]
