# Supplementary material for: AKT/FOXO1 axis links cross-talking of endothelial cell and pericyte in TIE2-mutated venous malformations
Source: Cell Commun Signal. 2020 Aug 31;18:139. doi: 10.1186/s12964-020-00606-w (PMC7457504; doi:10.1186/s12964-020-00606-w)
Supplement: Supplementary file 3 — Additional file 2. The mutant sequence of TIE2-L914F. [file 12964_2020_606_MOESM3_ESM.docx]

GGCCGTTTTTGGCTTTTTTGTTAGACGAAGCTTGGGCTGCAGGTCGACTCTAGAGGATCCCCGGGTACCGGTCGCCACCATGGACTCTTTAGCCAGCTTAGTTCTCTGTGGAGTCAGCTTGCTCCTTTCTGGAACTGTGGAAGGTGCCATGGACTTGATCTTGATCAATTCCCTACCTCTTGTATCTGATGCTGAAACATCTCTCACCTGCATTGCCTCTGGGTGGCGCCCCCATGAGCCCATCACCATAGGAAGGGACTTTGAAGCCTTAATGAACCAGCACCAGGATCCGCTGGAAGTTACTCAAGATGTGACCAGAGAATGGGCTAAAAAAGTTGTTTGGAAGAGAGAAAAGGCTAGTAAGATCAATGGTGCTTATTTCTGTGAAGGGCGAGTTCGAGGAGAGGCAATCAGGATACGAACCATGAAGATGCGTCAACAAGCTTCCTTCCTACCAGCTACTTTAACTATGACTGTGGACAAGGGAGATAACGTGAACATATCTTTCAAAAAGGTATTGATTAAAGAAGAAGATGCAGTGATTTACAAAAATGGTTCCTTCATCCATTCAGTGCCCCGGCATGAAGTACCTGATATTCTAGAAGTACACCTGCCTCATGCTCAGCCCCAGGATGCTGGAGTGTACTCGGCCAGGTATATAGGAGGAAACCTCTTCACCTCGGCCTTCACCAGGCTGATAGTCCGGAGATGTGAAGCCCAGAAGTGGGGACCTGAATGCAACCATCTCTGTACTGCTTGTATGAACAATGGTGTCTGCCATGAAGATACTGGAGAATGCATTTGCCCTCCTGGGTTTATGGGAAGGACGTGTGAGAAGGCTTGTGAACTGCACACGTTTGGCAGAACTTGTAAAGAAAGGTGCAGTGGACAAGAGGGATGCAAGTCTTATGTGTTCTGTCTCCCTGACCCCTATGGGTGTTCCTGTGCCACAGGCTGGAAGGGTCTGCAGTGCAATGAAGCATGCCACCCTGGTTTTTACGGGCCAGATTGTAAGCTTAGGTGCAGCTGCAACAATGGGGAGATGTGTGATCGCTTCCAAGGATGTCTCTGCTCTCCAGGATGGCAGGGGCTCCAGTGTGAGAGAGAAGGCATACCGAGGATGACCCCAAAGATAGTGGATTTGCCAGATCATATAGAAGTAAACAGTGGTAAATTTAATCCCATTTGCAAAGCTTCTGGCTGGCCGCTACCTACTAATGAAGAAATGACCCTGGTGAAGCCGGATGGGACAGTGCTCCATCCAAAAGACTTTAACCATACGGATCATTTCTCAGTAGCCATATTCACCATCCACCGGATCCTCCCCCCTGACTCAGGAGTTTGGGTCTGCAGTGTGAACACAGTGGCTGGGATGGTGGAAAAGCCCTTCAACATTTCTGTTAAAGTTCTTCCAAAGCCCCTGAATGCCCCAAACGTGATTGACACTGGACATAACTTTGCTGTCATCAACATCAGCTCTGAGCCTTACTTTGGGGATGGACCAATCAAATCCAAGAAGCTTCTATACAAACCCGTTAATCACTATGAGGCTTGGCAACATATTCAAGTGACAAATGAGATTGTTACACTCAACTATTTGGAACCTCGGACAGAATATGAACTCTGTGTGCAACTGGTCCGTCGTGGAGAGGGTGGGGAAGGGCATCCTGGACCTGTGAGACGCTTCACAACAGCTTCTATTGGACTCCCTCCTCCAAGAGGTCTAAATCTCCTGCCTAAAAGTCAGACCACTCTAAATTTGACCTGGCAACCAATATTTCCAAGCTCGGAAGATGACTTTTATGTTGAAGTGGAGAGAAGGTCTGTGCAAAAAAGTGATCAGCAGAATATTAAAGTTCCAGGCAACTTGACTTCGGTGCTACTTAACAACTTACATCCCAGGGAGCAGTACGTGGTCCGAGCTAGAGTCAACACCAAGGCCCAGGGGGAATGGAGTGAAGATCTCACTGCTTGGACCCTTAGTGACATTCTTCCTCCTCAACCAGAAAACATCAAGATTTCCAACATTACACACTCCTCGGCTGTGATTTCTTGGACAATATTGGATGGCTATTCTATTTCTTCTATTACTATCCGTTACAAGGTTCAAGGCAAGAATGAAGACCAGCACGTTGATGTGAAGATAAAGAATGCCACCATCACTCAGTATCAGCTCAAGGGCCTAGAGCCTGAAACAGCATACCAGGTGGACATTTTTGCAGAGAACAACATAGGGTCAAGCAACCCAGCCTTTTCTCATGAACTGGTGACCCTCCCAGAATCTCAAGCACCAGCGGACCTCGGAGGGGGGAAGATGCTGCTTATAGCCATCCTTGGCTCTGCTGGAATGACCTGCCTGACTGTGCTGTTGGCCTTTCTGATCATATTGCAATTGAAGAGAGCAAATGTGCAAAGGAGAATGGCCCAAGCCTTCCAAAACGTGAGGGAAGAACCAGCTGTGCAGTTCAACTCAGGGACTCTGGCCCTAAACAGGAAGGTCAAAAACAACCCAGATCCTACAATTTATCCAGTGCTTGACTGGAATGACATCAAATTTCAAGATGTGATTGGGGAGGGCAATTTTGGCCAAGTTCTTAAGGCGCGCATCAAGAAGGATGGGTTACGGATGGATGCTGCCATCAAAAGAATGAAAGAATATGCCTCCAAAGATGATCACAGGGACTTTGCAGGAGAACTGGAAGTTCTTTGTAAACTTGGACACCATCCAAACATCATCAATCTCTTAGGAGCATGTGAACATCGAGGCTACTTGTACCTGGCCATTGAGTACGCGCCCCATGGAAACCTTCTGGACTTCTTCCGCAAGAGCCGTGTGCTGGAGACGGACCCAGCATTTGCCATTGCCAATAGCACCGCGTCCACACTGTCCTCCCAGCAGCTCCTTCACTTCGCTGCCGACGTGGCCCGGGGCATGGACTACTTGAGCCAAAAACAGTTTATCCACAGGGATCTGGCTGCCAGAAACATTTTAGTTGGTGAAAACTATGTGGCAAAAATAGCAGATTTTGGATTGTCCCGAGGTCAAGAGGTGTATGTGAAAAAGACAATGGGAAGGCTCCCAGTGCGCTGGATGGCCATCGAGTCACTGAATTACAGTGTGTACACAACCAACAGTGATGTATGGTCCTATGGTGTGTTACTATGGGAGATTGTTAGCTTAGGAGGCACACCCTACTGCGGGATGACTTGTGCAGAACTCTACGAGAAGCTGCCCCAGGGCTACAGACTGGAGAAGCCCCTGAACTGTGATGATGAGGTGTATGATCTAATGAGACAATGCTGGCGGGAGAAGCCTTATGAGAGGCCATCATTTGCCCAGATATTGGTGTCCTTAAACAGAATGTTAGAGGAGCGAAAGACCTACGTGAATACCACGCTTTATGAGAAGTTTACTTATGCAGGAATTGACTGTTCTGCTGAAGAAGCGGCCGGTATGGACTACAAGGATGACGATGACAAGGATTACAAAGACGACGATGATAAGGACTATAAGGATGATGACGACAAATGAGCTAGCCTGTGGAATGTGTGTCAGTTAGGGTGTGGAAAGTCCCCAGGCTCCCCAGCAGGCAGAAGTATGCAAAGCATGCATCTCAATT

Green marks the mutant amino acid.
